# Human scattered tubular cells represent a heterogeneous population of glycolytic dedifferentiated proximal tubule cells

**DOI:** 10.1002/path.6029

**Published:** 2022-12-19

**Authors:** Jennifer Eymael, Martijn van den Broek, Laura Miesen, Valerie Villacorta Monge, Bartholomeus T van den Berge, Fieke Mooren, Vicky Luna Velez, Jelmer Dijkstra, Meyke Hermsen, Péter Bándi, Michiel Vermeulen, Saskia de Wildt, Brigith Willemsen, Sandrine Florquin, Roy Wetzels, Eric Steenbergen, Rafael Kramann, Marcus Moeller, Michiel F Schreuder, Jack FM Wetzels, Johan van der Vlag, Jitske Jansen, Bart Smeets

**Affiliations:** ^1^ Department of Pathology, Radboud Institute for Molecular Life Sciences Radboud University Medical Center Nijmegen The Netherlands; ^2^ Department of Pediatric Nephrology, Radboud Institute for Molecular Life Sciences Radboud University Medical Center, Amalia Children's Hospital Nijmegen The Netherlands; ^3^ Department of Nephrology, Radboud Institute for Molecular Life Sciences Radboud University Medical Center Nijmegen The Netherlands; ^4^ Department of Molecular Biology Radboud Institute for Molecular Life Science Nijmegen The Netherlands; ^5^ Department of Pharmacology and Toxicology Radboud Institute for Molecular Life Science Nijmegen The Netherlands; ^6^ Department of Pathology, Amsterdam UMC University of Amsterdam Amsterdam The Netherlands; ^7^ Amsterdam Institute for Infection and Immunology Amsterdam The Netherlands; ^8^ Division of Nephrology and Clinical Immunology RWTH Aachen University Aachen Germany; ^9^ Institute of Experimental Medicine and Systems Biology RWTH Aachen University Aachen Germany; ^10^ Department of Internal Medicine, Nephrology and Transplantation Erasmus Medical Center Rotterdam The Netherlands; ^11^ Department of Nephrology Radboud Institute for Health Sciences, Radboud University Medical Center Nijmegen The Netherlands

**Keywords:** scattered tubular cells, proximal tubule, kidney regeneration, acute kidney injury, (mal‐)adaptive repair

## Abstract

Scattered tubular cells (STCs) are a phenotypically distinct cell population in the proximal tubule that increase in number after acute kidney injury. We aimed to characterize the human STC population. Three‐dimensional human tissue analysis revealed that STCs are preferentially located within inner bends of the tubule and are barely present in young kidney tissue (<2 years), and their number increases with age. Increased STC numbers were associated with acute tubular injury (kidney injury molecule 1) and interstitial fibrosis (alpha smooth muscle actin). Isolated CD13^+^CD24^−^CD133^−^ proximal tubule epithelial cells (PTECs) and CD13^+^CD24+ and CD13^+^CD133^+^ STCs were analyzed using RNA sequencing. Transcriptome analysis revealed an upregulation of nuclear factor κB, tumor necrosis factor alpha, and inflammatory pathways in STCs, whereas metabolism, especially the tricarboxylic acid cycle and oxidative phosphorylation, was downregulated, without showing signs of cellular senescence. Using immunostaining and a publicly available single‐cell sequencing database of human kidneys, we demonstrate that STCs represent a heterogeneous population in a transient state. In conclusion, STCs are dedifferentiated PTECs showing a metabolic shift toward glycolysis, which could facilitate cellular survival after kidney injury. © 2022 The Authors. *The Journal of Pathology* published by John Wiley & Sons Ltd on behalf of The Pathological Society of Great Britain and Ireland.

## Introduction

Acute kidney injury (AKI) is a common clinical problem, estimated to occur in one in five adults and one in three children that are hospitalized with acute illness, and is associated with high morbidity and mortality rates [1, 2]. AKI can be defined as an abrupt decrease in glomerular filtration, in most cases caused by acute ischemic and/or toxic insults with the proximal tubule as the main site of injury, characterized by a loss of tubular cell function. Previously, it was shown that the proximal tubule has the capacity to regenerate and regain its function, depending on the degree of injury [3]. Despite the regenerative capacity of the tubules, kidney function does not always return to baseline level after repair, which can be indicative of progression to chronic kidney disease (CKD).

Scattered tubular cells (STCs) have a scattered distribution throughout the proximal tubule of normal human kidneys, show a less‐differentiated phenotype, and may play a role in (mal‐)adaptive repair processes [4, 5, 6, 7, 8]. Previously, we and others showed that STCs express the progenitor markers CD133 and CD24, have a higher proliferation index, become more numerous after AKI, and are more resistant to hypoxic injury [4, 6, 7, 9, 10, 11]. Lineage tracing experiments using mouse models of AKI indicated that STCs are not a fixed progenitor cell population but a transient phenotype that can arise from any proximal tubule epithelial cell (PTEC) after injury [5, 12]. However, debate is ongoing about whether STCs are progenitor/stem cells or if these cells are dedifferentiated PTECs [4, 5, 6, 7, 13, 14].

Next to tubular regeneration, STCs have also been linked to processes promoting failed repair. Earlier studies using mouse models showed that a subpopulation of injured dedifferentiated PTECs become arrested at the G2/M cell cycle phase and adopt a senescence‐associated secretory phenotype that may drive inflammation and fibrosis in the kidney. Single‐cell transcriptome analysis of PTEC subpopulations at different time points during ischemia–reperfusion injury (IRI) revealed a ‘failed‐repair’ population [15].

The majority of studies contributing to our knowledge of STCs were performed using animal models. However, the mouse findings may not be directly translatable to humans because STCs are only detected during injury and are absent under healthy conditions in mice. Also, the cell phenotype can be associated with a certain disease stage since disease processes start at a single time point, whereas in human nephrectomies the still unknown trigger may be chronic and cells may therefore be in different stages of injury and repair. Therefore, this study aimed to characterize the human STC population to gain a better understanding of their origin, location, heterogeneity, and function using immunohistochemical, immunofluorescence, and transcriptome analysis of human samples.

## Materials and methods

### Ethical statement and renal tissue

All experimental protocols were approved by the local ethics committee of the Radboud University Medical Center, Nijmegen, the Netherlands (CMO 2017‐3652), within the remit of the Medical Research Involving Human Subjects Act (WMO). All experiments were performed in accordance with the relevant guidelines and regulations. For marker analysis and isolation of PTECs as well as STCs, the unaffected pole of normal human renal cortex tissue from tumor nephrectomies was used. For the distribution analysis of STCs, normal human kidney tissue was obtained from tumor nephrectomies using the unaffected pole of 37 patients (48–85 years). Additionally, 29 kidney tissue samples were obtained from kidney transplant biopsies from donor kidneys prior to transplantation (age 23–76). Samples of 41 kidney tissues were obtained from autopsy (age 0–61 years). The paraffin samples consisted of postmortem autopsy kidney samples and surgical adult kidney samples from the Erasmus MC Tissue Bank, Rotterdam, the Netherlands. Tissues, which were selected for having no renal abnormalities in pathology and primary diagnosis, were procured at the time of autopsy within 48 h after death. In addition, all autopsy tissue was evaluated for autolysis and only included when the material had no or only mild autolysis. In total, autopsy tissue from seven patients was excluded, whereas 41 had no or only mild autolysis and were included. The Erasmus MC Research Ethics Board waived the need for formal ethics approval according to the Dutch Law on Medical Research in Humans. Pediatric tissue was collected when parental written informed consent for both autopsy and the explicit use of the tissue for research was present; adult tissue was collected within a nonobjection clause.

### Immunofluorescence and immunohistochemical staining

Procedures were performed according to [16]; see Supplementary materials and methods for details. For formalin‐fixed, paraffin‐embedded (FFPE) tissue, STC markers were previously validated by costaining with CD24 and/or CD133 [4].

### Slide registration and 3D tissue analysis

After immunohistochemical staining of consecutive slides of renal cortex tissue, light microscopic images of the whole tissue slides were made and digitized using the Panoramic P250 Flash II tissue scanner (3DHistech, camera CIS VCC/FC60FR19CL, software version 1.22.0.67865, Budapest, Hungary). The digitized slides were registered using the noncommercially available software HistokatFusion (Fraunhofer MEVIS lab, Bremen, Germany). The registration consisted of a prealignment, a parametric registration computed on coarse resolution, and a nonlinear, precise registration [17]. In all three steps the normalized gradient field distance, which measures the alignment of image gradients, was minimized [18]. The nonlinear registration adds a curvature regularizer to the distance term to favor smooth deformations without foldings [19]. After registration of the slides, 10 regions of interest were randomly selected and serial images were created using an automated slide analysis platform (ASAP, version 1.9, open‐source software). The number of STCs present as singlets or present in groups, as well as the number of STCs present at the inner bends of the proximal tubule, was counted. Three‐dimensional (3D) reconstructions were further created using the ImageJ 3D viewer plugin.

### Image analysis

For STC distribution analysis, eight random images (×20, fluorescence microscope) were taken of each patient tissue section stained for aquaporin‐1 (AQP1), phosphofructokinase‐platelet (PFKP), and nuclear counterstaining. The number of STCs was manually scored by two independent researchers. The number of PTECs was automatically counted using ImageJ (version 1.51) as outlined in the supplementary material (Supplementary materials and methods, supplementary material, Figure S1). As a result, the percentage of STCs (PFKP+) inside the PTECs (AQP1+) was calculated. For alpha‐smooth muscle actin (α‐SMA) analysis, five random images were taken of each patient tissue section (×10). The percentage of α‐SMA was calculated using the tissue sample surface area and the surface area of α‐SMA expression inside this area. This was automatically determined using ImageJ (version 1.51), as outlined in Supplementary materials and methods.

### Tissue preparation for FACS

Human renal cortex tissue from the unaffected pole of kidneys after tumor nephrectomy was cut into small pieces (≤1 mm) and further processed using enzymatic digestion and manual dissociation (Supplementary materials and methods). The isolated cell pellet was resuspended in 1 ml PBS containing 1% BSA (v/v, Sigma‐Aldrich, Zwijndrecht, the Netherlands), and antibodies against CD133, CD24, and CD13 (supplementary material, Table S1) were used to stain PTECs and STCs. Antibody incubation was performed for 1 h at 4 °C in the dark. The pellet was washed three times with PBS and centrifuged at 1,500 × *g* for 5 min, supernatant was removed, and the pellet was resuspended in PBS. This cell suspension was rinsed through a cell strainer (70 μm) and collected for cell sorting using the strategy described in Supplementary materials and methods.

### Bulk RNA sequencing

#### RNA isolation

RNA was isolated according to the PicoPure RNA Isolation Kit (Thermo Fisher Scientific, Breda, the Netherlands) manufacturer protocols. Samples were eluted in 11 μl elution buffer, and the purification column was incubated for 1 min at room temperature and centrifuged for 1 min at 1,000 × *g* followed by 1 min at 16,000 × *g*. The RNA sample was stored at −80 °C.

#### Bulk RNA‐sequencing sample preparation

Total RNA was used for library preparation using the RNA HyperPrep Kit with RiboErase (KAPA Biosystems, Wilmington, MA, USA). A fragmentation step was carried out for 6.5 min, and NextFlex DNA barcodes (Bioo Scientific, Austin, TX, USA) were used for adapter ligation. Libraries were amplified using 15 amplification cycles. Library concentration was measured using the dsDNA Fluorescence Quantification Assays (DeNovix, Wilmington, DE, USA), and library size was determined using the BioAnalyzer High Sensitivity DNA Kit (Agilent, Amstelveen, the Netherlands). Sequencing was performed using an Illumina NextSeq 500, and 50‐bp paired‐end reads were generated.

### RNA sequencing bioinformatic analysis

A workflow similar to that described in [16] was used; please see Supplementary materials and methods and supplementary material, Figure S2, for a detailed description.

### Statistical analysis

Statistical analysis was performed using GraphPad Prism (version 8.1.2, San Diego, CA, USA). Two‐tailed unpaired *t*‐tests were performed in the 3D analysis of STCs. A linear regression analysis was performed to analyze the number of STCs in relation to age and α‐SMA expression. A two‐tailed unpaired *t*‐test (Mann–Whitney *U* test) was performed to determine the association between kidney injury molecule 1 (KIM‐1) expression and the number of STCs. A one‐way ANOVA with Bonferroni's multiple comparison test was used to analyze the number of STCs in relation to age clusters. To determine the statistical difference between males and females, an unpaired *t*‐test (two‐tailed) was applied.

## Results

### STCs are mainly present in groups at inner bends of proximal tubule

**Figure 1:**
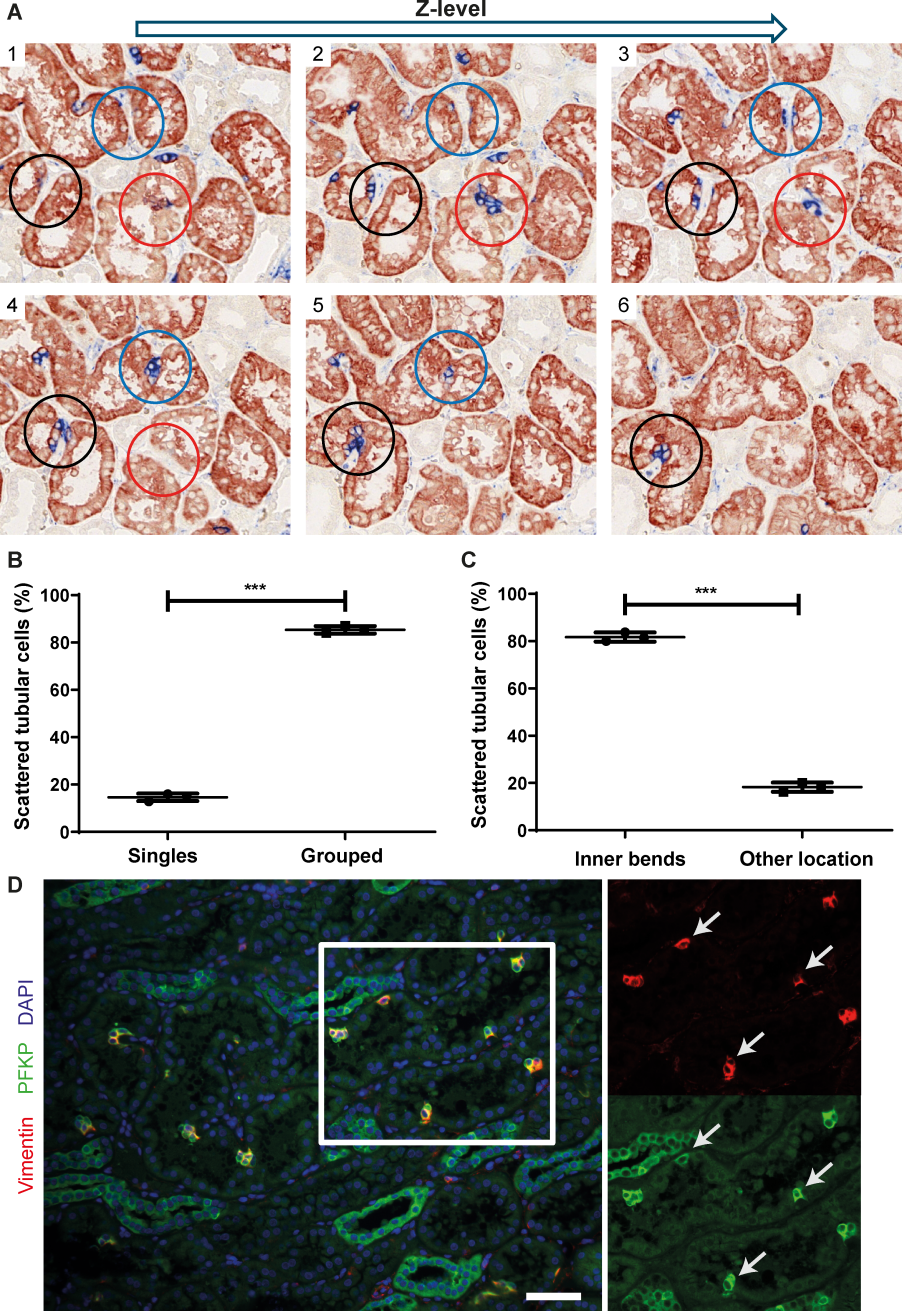
STCs are mainly present in groups located at the inner bends of the proximal tubule. (A) Immunohistochemistry staining for AQP‐1 (red) as PTEC marker and vimentin (blue) as STC marker. Microscopic images 1–6 show staining of serial slides of 2 μm from high *z*‐plane (image 1) to low *z*‐plane (image 6). The blue, black, and red circles show areas in which STCs can be detected in groups located at inner bends of the proximal tubule. Manual scoring of the STCs was performed in three different normal human tissues for 10 randomly chosen cortex areas of 20 *z*‐planes of serial slides per kidney. See supplementary material, Video S1, for a 3D reconstruction. (B) Percentage of STCs present as single cells or in groups of more than two cells in each normal human kidney. (C) Percentage of STCs at inner bends of proximal tubule and located elsewhere inside proximal tubule in each normal human kidney. (D) Immunofluorescence staining of normal human kidney for vimentin (red) and PFKP (green). STCs show co‐expression of both markers (white arrows). Scale bar: 50 μm. ***p < 0.0001, unpaired two‐tailed *t*‐test. AQP‐1, aquaporin‐1; PTEC, proximal tubule epithelial cell; STC, scattered tubular cell; PFKP, phosphofructokinase‐platelet.

The term STC refers to the observed distribution of the cells scattered within the human kidney cortex. We questioned whether the STCs were distributed randomly or if there was a structural location (niche) harboring STCs, similar to permanent stem or progenitor cell niches described in other organs [20]. We performed 3D tissue analysis, which enabled accurate analysis of STC tissue distribution, and we determined that STCs were located at the inner bends of the proximal tubule and located in groups consisting of two or more (up to 12) STCs (Figure 1A–C; supplementary material, Video S1). Although some STCs seemed to be in a straight part of the proximal tubule in single sections (Figure 1A), 3D reconstruction of aligned serial sections often revealed a bend in a higher or lower *z*‐level in the tissue (supplementary material, Video S1). These results demonstrated that the location of STCs was not random; there was a preferred location within the sharp inner bends of the tubule.

Additionally, we identified PFKP as a novel STC marker, confirmed by co‐expression with vimentin (Figure 1D). STCs stained for PFKP or vimentin were observed in AQP1, glucose transporter 2 (GLUT2), and megalin (LRP2) expressing proximal tubule segments (supplementary material, Figure S3A–C*). GLUT2 and LRP2 are expressed in the proximal tubule segments S1 and S2 [21, 22], whereas AQP1 is highly expressed in the proximal straight tubules and shows a less abundant expression in the convoluted part of the proximal tubule [23]. These data indicate the presence of STCs in all segments of the proximal tubule.

### STC number increases with age and kidney injury

**Figure 2:**
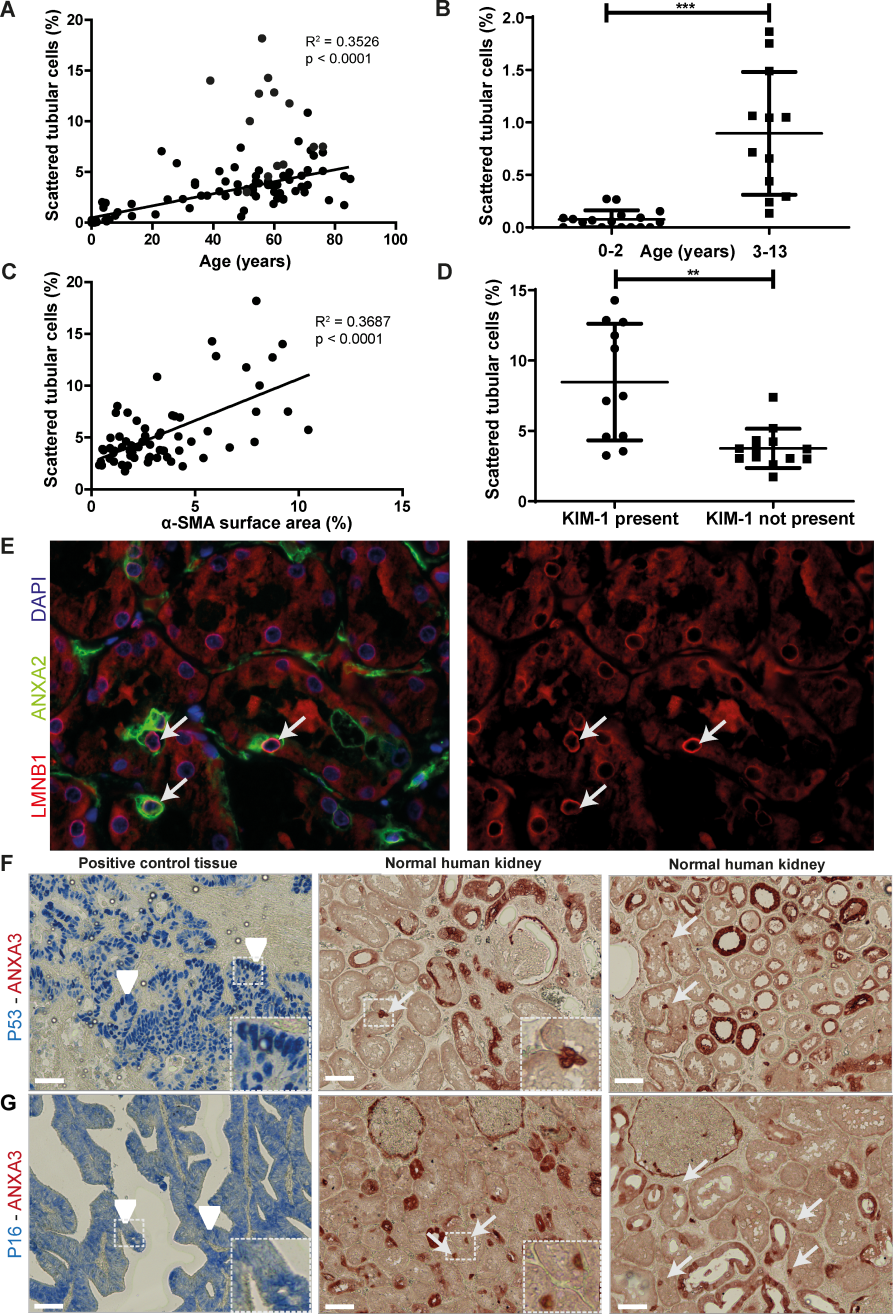
STC number increases in renal aging and injury but cannot be associated with cellular senescence. (A) Regression analysis of STC number versus age in normal human kidney tissue from tumor nephrectomies using the unaffected pole of 37 patients (48–85 years), 29 renal transplant biopsies from donor kidneys prior to transplantation (age 23–76) and 41 kidney tissues obtained from autopsy (age 0–61 years). (B) STC numbers in children aged 0–13. Children aged 0–2 years show mean 0.076 ± SD 0.09 STCs (*N* = 17), those aged 3–13 years show mean 0.90 ± SD 0.59, (*N* = 12). (C) Regression analysis of STC number and α‐SMA expression surface area in normal human kidneys from tumor nephrectomy. (D) Relationship between KIM‐1 and number of STCs in normal human kidney tissue derived from tumor nephrectomy. (E) Representative immunofluorescence LMNB1 (red) and STC marker ANXA2 (green). A normal to high level of LMNB1 around the nucleus (blue, DAPI staining) is found in STCs (arrow). (F) Immunohistochemistry for p53 (blue) and ANXA3 (red). Arrowheads show p53 in positive control tissue, arrows indicate STCs. (G) p16 immunohistochemistry (blue) and ANXA3 (red). Arrowheads indicate positive control tissue, and arrows show STCs in normal human kidneys. Scale bar: (F and G) 50 μm. **p < 0.001, ***p < 0.0001. STC, scattered tubular cell; α‐SMA, alpha‐smooth muscle actin; KIM‐1, kidney injury molecule‐1; LMNB1, lamin B1; ANXA2, annexin A2; ANXA3, annexin A3.

In our 3D analysis, STCs seemed to be a structural part of the inner bends of the proximal tubule, suggesting these bends might be a niche harboring STCs. We hypothesized that if STCs were dedifferentiated PTECs, they would not be present in young kidney tissue and increase with age, whereas a pre‐existing progenitor cell population would be present and might decrease with age. Analysis revealed that the number of STCs (PFKP+, AQP1+) was negligible in children 0–2 years old, whereas the number of STCs increases with age, arguing in favor of a dedifferentiated PTEC population rather than a pre‐existing progenitor cell population (*R* ^2^ = 0.3526, Figure 2A,B). For all age groups, samples showing α‐SMA staining of more than 5% of the tissue surface area were excluded for the age group clustering because they could be classified as pathologically suspicious, showing marked interstitial fibrosis (supplementary material, Figure S4A). STC numbers did not differ between male and female but did show a difference between age groups (child‐young adults, adults, middle‐aged, and elderly) (supplementary material, Figure S4B,C).

To study the relationship between the number of STCs and kidney injury, we analyzed the amount of fibrosis (α‐SMA^+^) present in normal human kidney tissue and the presence of acute tubular injury by the presence of KIM‐1. An increased number of STCs (PFKP^+^) was found in tissues with increased fibrosis (*R* ^2^ = 0.3893) or KIM‐1 presence (Figure 2C,D, supplementary material, Figure S4D–F). In summary, these results suggest a relation between the number of STCs and interstitial fibrosis as well as tubular injury and indicate that the presence of STCs is related to kidney injury.

### STCs show no marker expression associated with cellular senescence

To test whether STCs express markers associated with cell senescence, immunostaining of three different normal human kidney tissues and three sections per tissue was performed. Lamin B1 (LMNB1) is located around the nucleus and degraded in senescent cells. In annexin A2 (ANXA2)‐positive STCs, normal LMNB1 surrounding the nucleus can be found (Figure 2E). Tumor suppressors p53 and p16, both known to be upregulated in senescent cells, were not detected in STCs (Figure 2F,G), suggesting that STCs are not senescent cells.

### Comparative transcriptome profiling of sorted PTEC and STC

**Figure 3:**
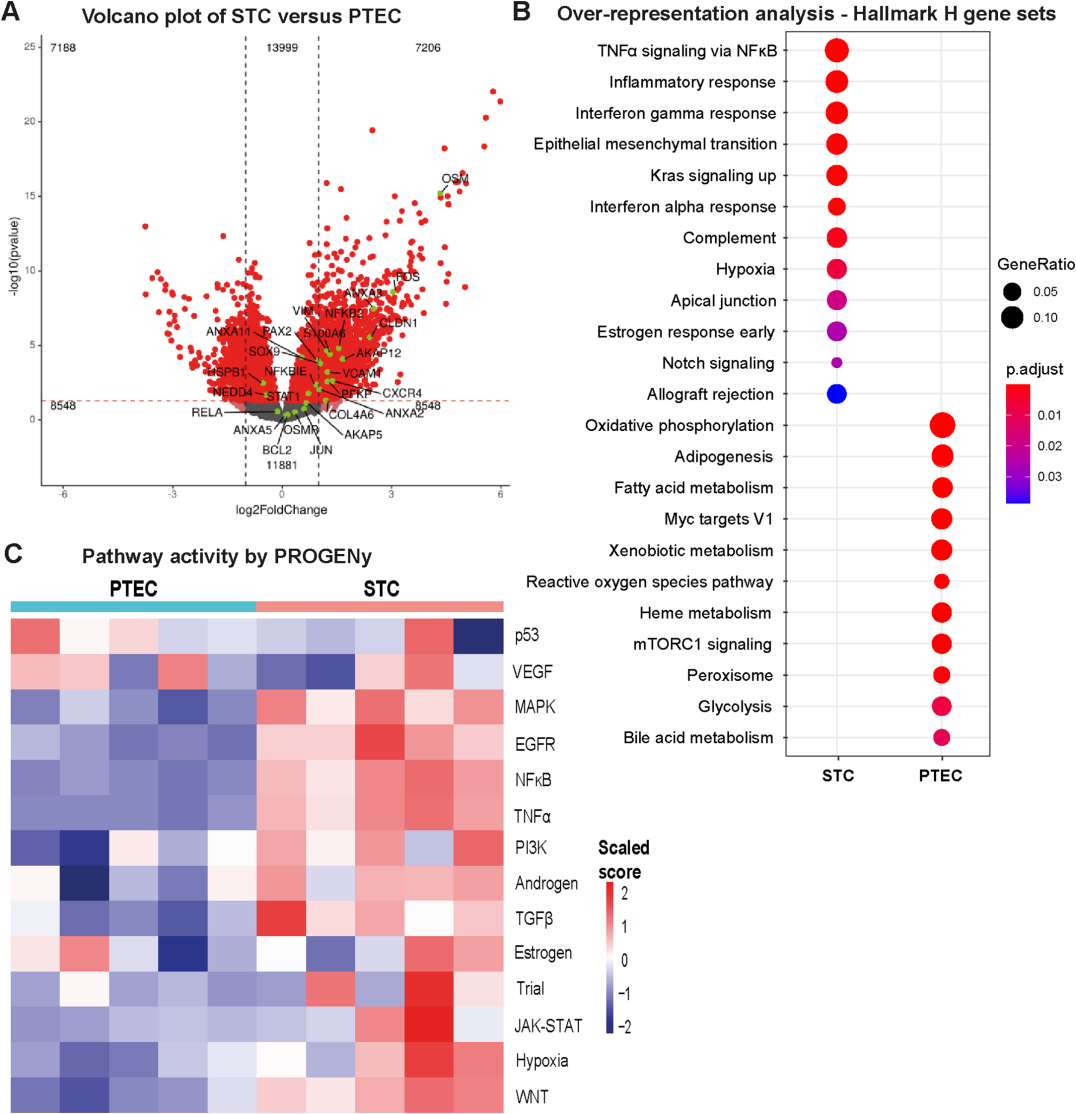
Transcriptome and pathway analysis shows significant differences between STCs and proximal tubular cells. (A) Volcano plot of PTEC versus STC transcriptomes; *y*‐axis indicates statistical significance (−log10(*p* value)) and magnitude of change on *x*‐axis (log2FoldChange). Statistically significant different genes are highlighted in red. Known STC markers (based on protein expression [2]) are highlighted in green. (B) Overrepresentation analysis using Hallmark H gene sets for significantly upregulated genes in PTEC and STC population. (C) Pathway activity by PROGENy in PTEC and STC population for the 14 included pathways. PTEC, proximal tubular epithelial cell; STC, scattered tubular cell.

To compare the transcriptomic profile of the STC (CD13+CD24+ and CD13+CD133+) population to the PTEC (CD13+CD24‐CD133‐) population, we performed bulk RNA sequencing of the sorted populations (supplementary material, Figure S5A–D). Exploratory data analysis showed that PTECs and STCs from patients 6 and 7 deviated from those of the other patients, so these patients were not used for downstream analyses (supplementary material, Figure S5E,F). Next, differential gene expression (DGE) analysis was performed and confirmed the successful isolation of PTECs and STCs (Figure 3A). In addition, genes that were significantly upregulated in the STCs compared to PTECs were also expressed at the protein level in human STCs, such as ANXA3 and A‐kinase anchoring protein 12 (AKAP12) (supplementary material, Figure S6A,B). Overrepresented terms in PTECs using Hallmark H gene sets are in line with normal PTEC functioning, for instance, PTECs have active mTORC1 signaling, reactive oxygen species pathway, and xenobiotic, heme, bile acid, and fatty acid metabolism (Figure 3B) [24, 25, 26, 27].

The majority of overrepresented terms and pathway activities in the STC population are related to inflammatory and fibrosis‐associated pathways, such as tumor necrosis factor alpha (TNFα) and nuclear factor kappa B (NFκB) signaling, interferon‐alpha (IFN‐α), and interferon‐gamma (IFN‐γ) (Figure 3B,C). Of note, STC showed pathways associated with repair are active, such as epidermal growth factor receptor (EGFR) and Wnt signaling [28, 29].

### STCs show a metabolic switch toward glycolysis

**Figure 4:**
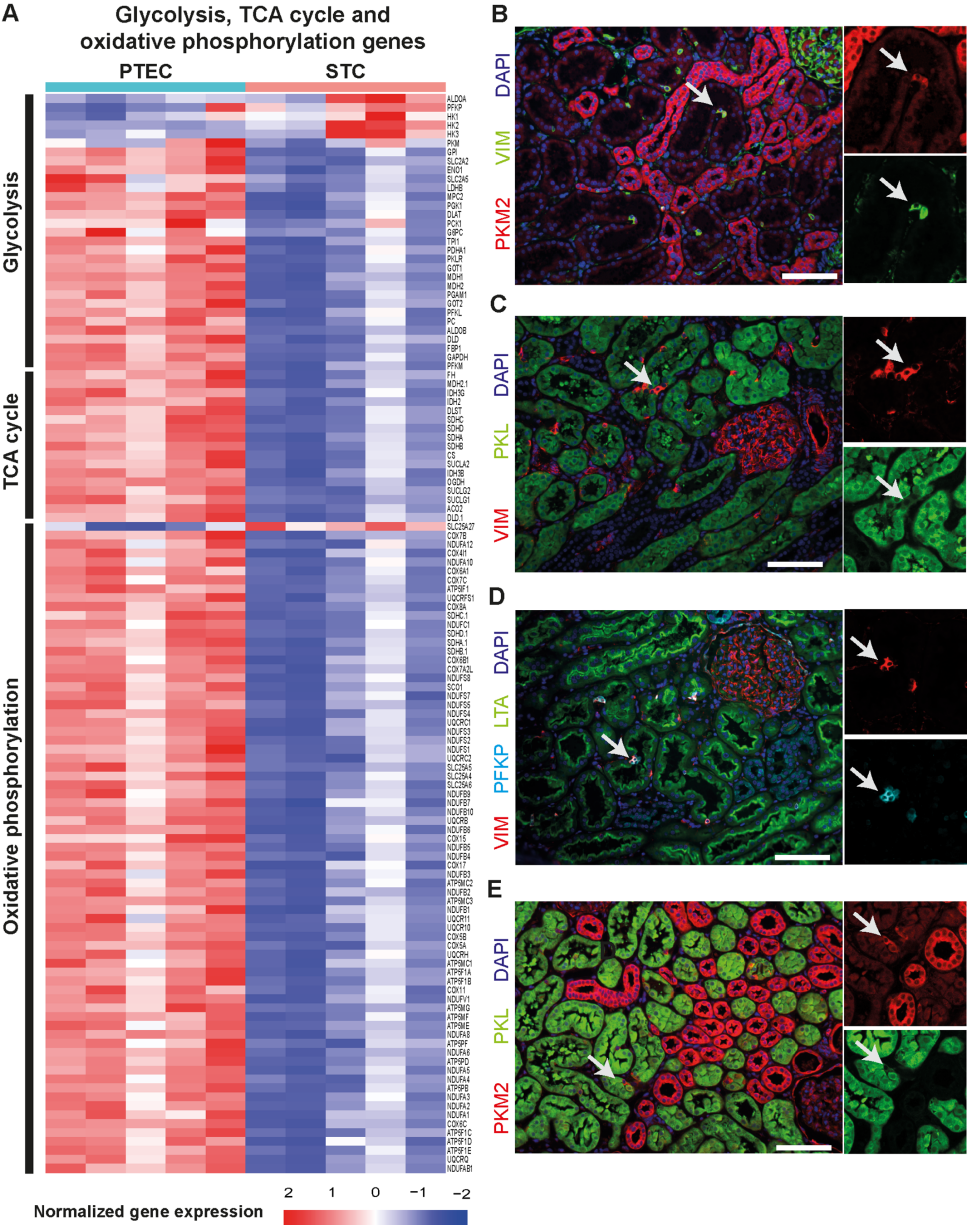
Downregulation of mitochondrial respiration in STCs. (A) Heatmap of genes involved in glycolysis, electron transport chain, and TCA cycle (Krebs or citric acid cycle). Heatmaps show the row‐wise *z*‐score per sample per gene for PTECs versus STCs. (B) Immunofluorescence for PKM2 (red) and vimentin (green). (C) Immunofluorescence for vimentin (red) and PKL (green). Arrow indicates STCs. (D) Representative image of immunofluorescence for PFKP (cyan) and vimentin (red) in STCs (arrow) located inside proximal tubule (LTA, green). (E) Double staining for PKM2 (red) and PKL (green). Arrow indicates STCs. Scale bars: (B–E) 100 μm. TCA, tricarboxylic acid; PTEC, proximal tubular epithelial cell; STC, scattered tubular cell; PKM2, pyruvate kinase M2; PKL, pyruvate kinase L; LTA, *Lotus tetragonolobus* lectin.

To further dissect the metabolic changes in STCs, we visualized the normalized gene expression of the oxidative phosphorylation, tricarboxylic acid (TCA) cycle, and glycolysis pathways. The majority of genes involved in these pathways are downregulated in STCs (Figure 4A). As previously described, electron microscopic images showed a high density of mitochondria in PTECs, whereas STCs showed nearly no mitochondria inside the cytoplasm (supplementary material, Figure S7A,B) [4], which is in accordance with the downregulation of genes involved in TCA cycle and oxidative phosphorylation. Although several genes of glycolysis are downregulated in STCs, genes of key regulators of glycolysis, such as hexokinase and phosphofructokinase (PFK), are upregulated (Figure 4A, supplementary material, Table S3). Furthermore, we observed a switch in the isoform expression of PFK and pyruvate kinase (PK). The PTECs of normal healthy kidneys showed exclusive expression of PK‐Liver(L), whereas STCs (vimentin^+^) expressed PK‐Muscle 2(M2) and PFK‐Platelet(P) (Figure 4D–G) [30]. RNA sequencing data showed downregulation of *PFKL* and *PKLR* and an upregulation of *PFKP* in STCs (supplementary material, Tables S3 and S4). Furthermore, two lactate transporters encoded by *SLC16A3* (monocarboxylate transporter 4) and *SLC16A7* (monocarboxylate transporter 2) were also significantly upregulated in STCs compared to PTECs (supplementary material, Table S3) [31]. Altogether, these results suggest a glycolytic switch in STCs compared to PTECs, although future research should focus on functional studies to confirm this glycolytic switch.

### STCs show a heterogeneous phenotype

**Figure 5:**
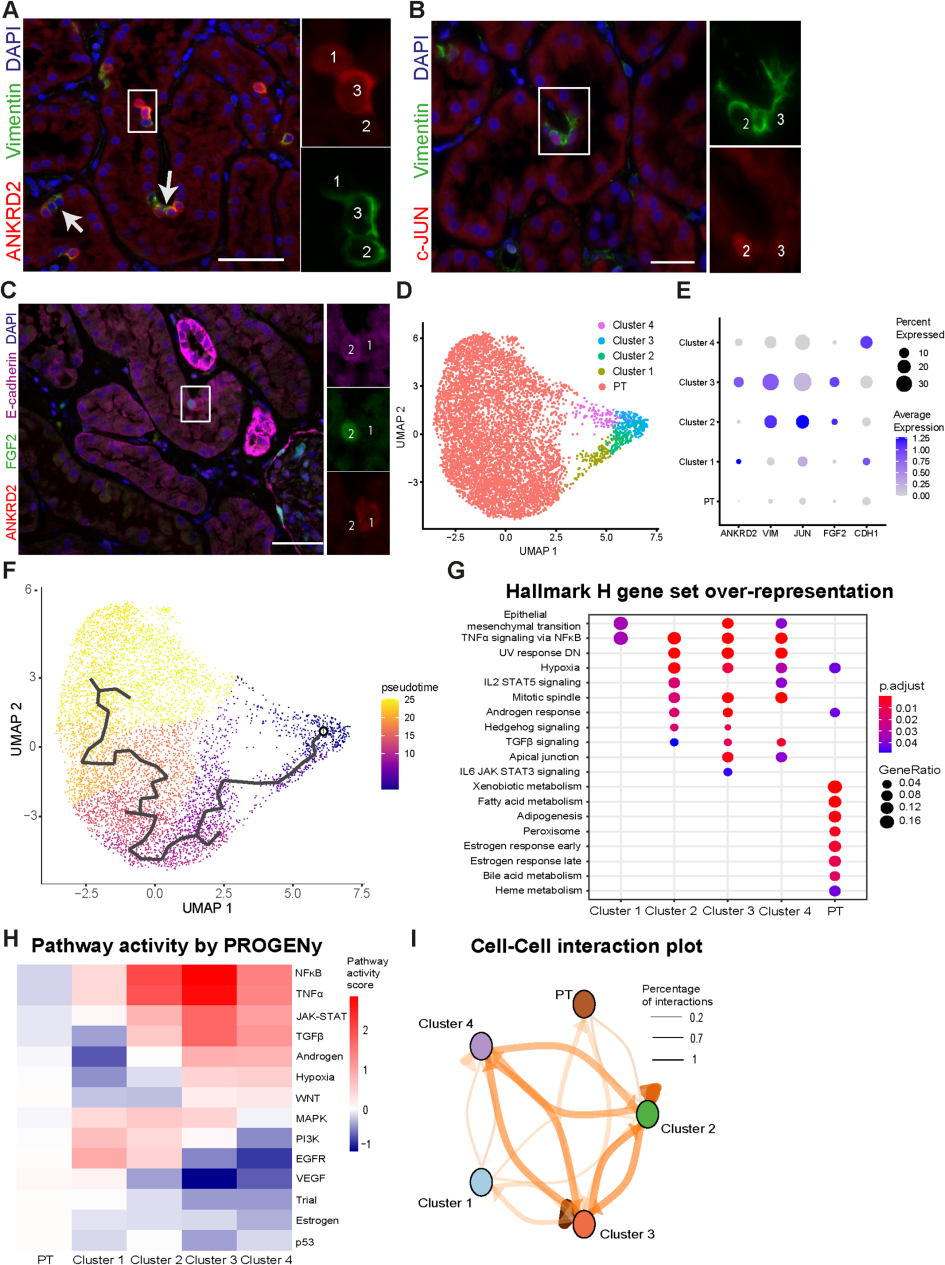
Single‐cell sequencing identifies multiple cell clusters that resemble STCs in normal human kidney. (A) Immunofluorescence staining for ANKRD2 (red) and vimentin (green). Cell 1 shows expression of ANKRD2 but not vimentin, whereas in Cell 2 vimentin is expressed but ANKRD2 is not. Both ANKRD2 and vimentin are detected in Cell 3. (B) Similar heterogeneity was observed for c‐JUN (red) and vimentin (green) and for (C) ANKRD2 (red), FGF2 (green), and E‐cadherin (magenta). (D) Isolated proximal tubule cluster with four subclusters of proximal tubule cells expressing *VCAM1* from an online dataset. (E) Dot plot of gene expression of immunofluorescent markers in (A–C). The clusters to which the cells belong are indicated in the immunofluorescent images, and, except for E‐cadherin, all other heterogeneous expression patterns are also observed in the single‐cell RNA sequencing dataset. (F) Pseudotime trajectory in isolated proximal tubules and VCAM1^+^ proximal tubule clusters as determined by Monocle. (G) Comparative overrepresentation analysis using Hallmark H gene sets from MSigDB for proximal tubule cells and PT_VCAM1 clusters. (H) Pathway activity per cell cluster using PROGENy. (I) Cell–cell interaction plot to visualize amount of ligand–receptor interactions between clusters. Line thickness reflects amount of ligand–receptor interactions. Scale bars: (A–C) 50 μm. ANKRD2, ankyrin repeat domain 2; FGF2, fibroblast growth factor 2; MSigDB, molecular signature database; STC, scattered tubular cell.

Heterogeneous expression of several STC markers was regularly observed in neighboring cells (Figure 5A–C). As such, STCs solely expressed ankyrin repeat domain 2 (ANKRD2), only vimentin, or both (Figure 5A). To resolve the heterogeneous profile and driving factors of STC subtypes, we used the whole human kidney single‐cell RNA‐seq dataset from Muto *et al* [32] and identified 4 clusters of STC (Figure 5D). We evaluated the markers ANKRD2, vimentin, c‐JUN, fibroblast growth factor 2 (FGF2), and E‐cadherin in these subclusters. We identified a similar heterogeneous expression of STC markers in the single‐cell dataset as observed on protein level for ANKRD2, vimentin, c‐JUN, and FGF2 (Figure 5A–C,E).

Next, we performed pseudotime analysis, a measure of how far a cell is in a biological process (Figure 5F, supplementary material, Table S5). Nearly all STC markers are significantly associated with pseudotime and are highly expressed in especially STC subclusters 2 and 3 (supplementary material, Figure S8A) [4]. Also, several transporters, including sodium/glucose cotransporter 2 (SGLT2), organic anion transporter 1 (OAT1), and cubilin (CUBN), that enable normal PTEC function are associated with pseudotime and are transiently lower expressed in STC subclusters (supplementary material, Figure S8A). However, STC cluster 4 has increased expression of transporters and decreased expression of STC markers compared to cluster 3, which might reflect a redifferentiation cluster toward PTECs.

By comparing the PT and STC subclusters from the single‐cell dataset with our bulk RNA samples, we could indicate their resemblance and confirm cell characteristics. PT cells showed similarly overrepresented gene sets (bile acid, heme, and fatty acid metabolism) as sorted PTECs, whereas STC subclusters had overrepresented gene sets (TNFα signaling *via* NFκB, apical junction, hypoxia, and epithelial mesenchymal transition), similarly to our sorted STCs (Figures 3B and 5G). We also observed a switch in PFK and PK isoforms between PT and STC clusters (supplementary material, Figure S8B). Using PROGENy, we found that similar pathways (NFκB, TNFα, TGFβ) are active in the STC subclusters compared to our sorted STCs (Figure 5H).

We observed heterogeneity in pathway activity, with clusters 1 and 2 being more active in repair pathways (PI3K and EGFR) and clusters 3 and 4 being more active in profibrotic and stress pathways (Wnt and hypoxia) (Figure 5H) [33]. We also observed heterogeneity in the ligand–receptor interactions; for instance, STC subclusters 2 and 3 are the only clusters that signal with the proximal tubule cells (Figure 5I). Also, the majority of ligand–receptor interactions between STC subclusters and PTs are *via* EGFR‐receptor family ligands and receptors (supplementary material, Table S6), suggesting an important role for EGFR signaling in STC and PTEC communication.

## Discussion

In this study, we aimed to characterize the origin and role of the human STC population. We showed that STCs are a dedifferentiated PTEC population since STCs are rarely present in young children and increase in number with age and injury. STCs are not senescent, which is a hallmark of cells contributing to maladaptive repair, and are therefore not likely to contribute to maladaptive repair. Transcriptome analysis using bulk RNA sequencing revealed an upregulation of inflammatory pathways in STCs, whereas metabolism and, especially, the TCA cycle and oxidative phosphorylation were downregulated, indicating that STCs showed a metabolic switch toward glycolysis. Furthermore, we could determine that STCs represent a heterogeneous population.

Since the origin and function of STCs are still debated, we analyzed the number of STCs in human kidney tissue in relation to age and kidney injury [7, 8, 14, 34, 35]. Progenitor and stem cells are known to be present in high numbers in younger tissue. Our results showed that STCs were hardly ever present in kidney tissue of patients 0–2 years of age, but their numbers increased with age. In line with our findings, the cells described by Hansson *et al* showed a similar morphology using electron microscopy, had the same STC marker expression (vimentin, CD24 and CD133), and showed an absence of mitochondria [36]. In contrast, that study showed no correlation between age and STC number, which is likely attributable to the relatively small tissue cohort used.

Furthermore, we showed that STCs were located not in a protective niche environment but primarily at the inner turns of the proximal tubule. We hypothesized that STCs were more likely to be exposed to stressors in the inner bends, such as shear stress, because fluid flow velocity is highest in the inner bend of a tubing system [37]. This localization was mentioned previously since STCs were described to be generally seen at the tubular plicae, where the proximal tubule makes hairpin turns [4, 36, 38]. Additionally, STC number correlated with increased kidney injury and tubulointerstitial fibrosis, supposedly due to an increased number of injured PTECs stimulating dedifferentiation to STCs. Altogether, these results support the hypothesis that STCs are dedifferentiated PTECs, as opposed to being a progenitor or stem cell population.

A second question regarding human STCs is whether they contribute to tubular regeneration or if the morphological and functional changes are irreversible, leading to cellular senescence, contributing to interstitial fibrosis and tubular atrophy [39, 40, 41, 42]. Although cellular senescence is linked to aging [43, 44], our results indicate that STCs are not cell cycle arrested or senescent since no expression of senescence‐associated markers was detected in STCs. These findings are in line with earlier studies, showing that STCs express markers associated with cell survival and proliferation, suggesting that STCs are able to proliferate and contribute to tubular repair [4, 6, 7]. A suggestion further supported by the finding that adult human kidney organoids are derived from a CD24+ proximal tubular cell population [45]. On the other hand, a recent study indicated that the endocycle may play a role in which cells expand in size to restore the integrity of the epithelium and express proliferation markers [46]. Therefore, it is still debated whether the observation of proliferation markers indicates dividing cells or cells in endocycle.

The RNA sequencing results, electron microscopy, and immunofluorescence staining indicated that STCs are glycolytic and rely less on mitochondrial respiration to obtain energy. STCs showed expression of both the PFKP and PKM2 isoform, instead of PKL and PFKL. The PKM2 dimer stimulates the conversion of pyruvate into lactate, whereas the tetramer of PKM2 stimulates normal entry of the TCA cycle after glycolysis and, thus, oxidative phosphorylation [47, 48]. Furthermore, these results do not support the suggestion that STCs actively proliferate to replace lost cells since metabolism is downregulated in such a way that the formation of new essential cellular components needed for cell division is absent. It was shown earlier in mice that STC‐like cells were the main proliferating cell population contributing to regeneration after ischemia‐reperfusion (IR) injury [5]. However, in humans, it was found that the few proliferating cells are STCs rather than PTECs, but most human STCs are not proliferating [4]. The observation that STCs show a heterogeneous phenotype, even within one cluster of cells, suggests a transient process of PTEC dedifferentiation and metabolic reprogramming, which could facilitate cell survival. STCs might be able to survive the hypoxic environment that is associated with AKI due to their glycolytic switch and are then able to proliferate and redifferentiate into functional PTECs again.

Our human STC samples showed STC marker expression similar to that of the human STC clusters we identified in the Muto *et al* database, and therefore we consider these cells to be the same [32]. Muto *et al* showed that their human STC (PT‐VCAM1) cluster resembled the failed‐to‐repair proximal tubule cell cluster (FR‐PT) identified in mice [32]. Indeed, both the two human STC populations and the murine FR‐PTs show increased pro‐inflammatory and profibrotic pathway activity and recently the FR‐PT population in mice was indicated to be more glycolytic since they have an increased lactate production [15, 32, 49]. However, the authors compared the entire human STC population to these mouse FR‐PTs, whereas we now identified several subclusters of human STCs with heterogeneous marker expression, on both the gene and protein levels, and pathway activity profiles. The heterogeneity of human STCs is crucial to unravel since some human STC subclusters have a different pathway activity profile and/or marker expression compared to mouse FR‐PTs. For example, STC cluster 1 shows lower pro‐inflammatory and profibrotic pathway activity, STC cluster 4 shows higher transporter gene expression (*i.e*. LRP2 and CUBN) important for normal PTEC function, and both STC clusters 1 and 2 show higher prosurvival EGFR activity [28]. Altogether, some human STCs might be more similar to FR‐PTs, whereas other STCs might still be able to recover or are in the process of recovering.

One of the heterogeneously active pathways that might be involved in STC formation and PTEC survival is the EGFR pathway, which was also highly active in the bulk RNA sequencing results of our STCs compared to PTECs. Since subcluster 1 resembles the proximal tubule cluster most and has EGFR activation that was not seen in subclusters 3 and 4, EGFR might activate a downstream cascade leading to the STC phenotype. A previous study in mice identified FOXM1 as an early induced transcription factor in injured PTECs, which was dependent on EGFR stimulation [28]. EGFR pathway activity may lead to KRAS signaling activation, which in turn can activate both MAPK and PI3K pathways [50]. This might lead to PFKP phosphorylation, which leads to β‐catenin stabilization and PKM2 binding to β‐catenin to activate Wnt signaling [51]. The PI3K pathway can then lead to NFκB activation and subsequent TNFα signaling. EGFR signaling can increase the expression of PKM2, which is extensively reviewed in cancer cells to promote tumor growth and might also contribute to the proliferative phenotype of injured PTECs [30, 52]. This possible role of EGFR calls into question anti‐EGFR therapy, which is indeed associated with many renal adverse events, such as electrolyte disturbances, and might be a ‘second hit’ for the development of AKI [53, 54]. Hence, the use of anti‐EGFR agents could interfere with STC pathways important for proximal tubular regeneration [55].

In conclusion, our results indicate that STCs are not a fixed progenitor cell population but are dedifferentiated PTECs. Even though STC number is related to age and renal injury, STCs are not senescent. Furthermore, STCs show a metabolic switch towards glycolysis, which could be facilitated by EGFR signaling and PKM2 expression and might be important for tubular cell survival. Importantly, STCs are a heterogeneous cell population. However, whether human STCs actively contribute to adaptive repair of the proximal tubule and whether these cells are able to redifferentiate into functional PTECs needs further investigation.

## Author contributions statement

JE and MB performed data acquisition, analysis, interpretation, a literature search, and figure generation, drafted the first version of the manuscript, and completed the final version of the manuscript. LM, VVM, BB, FM, BW, VLV, JD and RW carried out experiments and analyzed/interpreted data. MH and PB carried out computational experiments and data analysis. SW and SF provided crucial kidney samples and revised the manuscript. ES, RK, MM, MV, MFS and JW designed experiments and revised the manuscript. JV designed experiments, interpreted the data, and revised the manuscript. JJ and BS conceived the study and experiments, interpreted data, and aided in writing the second version of the manuscript and figure generation. All authors approved the final version of the submitted version.

## Supporting information


Supplementary materials and methods
**Figure S1.** Fiji workflow for automated counting of proximal tubular epithelial cells (PTECs)
**Figure S2.** Preprocessing of single‐cell sequencing database and subsequent clustering
**Figure S3.** Scattered tubular cells are present in all segments of proximal tubule
**Figure S4.** Scattered tubular cell numbers increase with age
**Figure S5.** Fluorescence‐activated cell sorting strategy and exploratory bulk RNA sequencing analysis
**Figure S6.** Immunohistochemical validation of STC markers upregulated in bulk RNA sequencing data
**Figure S7.** Scattered tubular cells show less mitochondria and a rudimentary brush border and basal labyrinth
**Figure S8.** Scattered tubular cell clusters exhibit different gene expression profiles
**Table S1.** Primary antibodies used for immunofluorescence and immunohistochemical staining
**Table S2.** Secondary antibodies used for immunofluorescence and immunohistochemical staining (referred to in supplementary material)

**Table S3.** deseq2_upregulated_Scattered_vs_Proximal

**Table S4.** deseq2_downregulated_Scattered_vs_Proximal

**Table S5.** Monocle_markers

**Table S6.** LR_interactions

**Video S1.** 3D reconstruction to study location of scattered tubular cells (vimentin in blue, AQP‐1 in red)

## Data Availability

For single‐cell sequencing analysis we used a dataset that is deposited on GEO with Accession No. GSE151302. The bulk RNA sequencing data are deposited on GEO with Accession No. GSE202824 (accessible at GSE202824). All scripts used for RNA sequencing analysis in RStudio are deposited on Zenodo and accessible using the following link: https://doi.org/10.5281/zenodo.6168927.
