# Supplementary material for: Human scattered tubular cells represent a heterogeneous population of glycolytic dedifferentiated proximal tubule cells
Source: J Pathol. 2022 Dec 19;259(2):149–62. doi: 10.1002/path.6029 (PMC10107692; doi:10.1002/path.6029)
Supplement: Supplementary file 1 — Supplementary materials and methods Figure S1. Fiji workflow for automated counting of proximal tubular epithelial cells (PTECs) Figure S2. Preprocessing of single‐cell sequencing database and subsequent clustering Figure S3. Scattered tubular cells are present in all segments of proximal tubule Figure S4. Scattered tubular cell numbers increase with age Figure S5. Fluorescence‐activated cell sorting strategy and exploratory bulk RNA sequencing analysis Figure S6. Immunohistochemical validation of STC markers upregulated in bulk RNA sequencing data Figure S7. Scattered tubular cells show less mitochondria and a rudimentary brush border and basal labyrinth Figure S8. Scattered tubular cell clusters exhibit different gene expression profiles Table S1. Primary antibodies used for immunofluorescence and immunohistochemical staining Table S2. Secondary antibodies used for immunofluorescence and immunohistochemical staining (referred to in supplementary material) [file PATH-259-149-s003.docx]

**Human scattered tubular cells represent a heterogeneous population of glycolytic dedifferentiated proximal tubule cells**

J Eymael, M van den Broek *et al*. *J Pathol* <https://doi.org/10.1002/path.6029>

**Supplementary materials and methods**

**Supplementary Figures S1–S8**

**Supplementary materials and methods**

Reference numbers refer to the main text list

**Immunofluorescence staining**

Formalin-fixed, paraffin-embedded tissue sections (4-μm) were deparaffinized using xylene and rehydrated. Antigen retrieval was performed using citrate buffer (pH 6, ScyTek Laboratories, Logan, UT, USA). Prior to primary antibody incubation, sections were incubated with normal goat serum (Vector Laboratories, Peterborough, UK) in PBS for 10 min. Incubation with the primary antibodies in 1% BSA (v/v, Sigma-Aldrich, Zwijndrecht, The Netherlands) in PBS was performed overnight at 4 °C (supplementary material, Table S1). The secondary antibodies were incubated for 2 hours at room temperature in the dark (supplementary material, Table S2). Slides were mounted with DAPI fluoromount G (Southern Biotech, Birmingham, AL, USA) and covered with a cover slip. Sections were examined using high-content fluorescence microscopy (Leica DMI6000B).

**Immunohistochemical staining**

Formalin-fixed, paraffin-embedded human renal tissue sections were deparaffinized using xylene and rehydrated. Antigen retrieval was performed in Tris/EDTA buffer pH 9 (VWR Chemicals, Amsterdam, The Netherlands). Endogenous peroxidase blocking was performed for 10 min in 0.3% hydrogen peroxide (v/v, Boom B.V., Drenthe, The Netherlands). Avidin and biotin blocking kit (Vector Laboratories) was used following the manufacturer’s instructions. Prior to primary antibody incubation, sections were incubated with normal horse serum (Vector laboratories) in PBS for 10 min. Primary antibody incubation was done for 2 h at room temperature (supplementary material, Table S1). Thereafter, sections were incubated with biotinylated horse anti-mouse antibody (supplementary material, Table S2) for 30 min at room temperature, followed by incubation with ABC*AP solution (Vector Laboratories), following the manufacturer’s instructions. After washing, tissue sections were incubated with StayBlue AP developing solution (Abcam, Cambridge, UK) for 5 min. Incubation with a second primary antibody was performed overnight at 4 °C (supplementary material, Table S1), followed by incubation with HRP-conjugated secondary antibody (supplementary material, Table S2) for 30 min at room temperature. Color development used AEC substrate (Abcam). Tissue sections were mounted with quick D mounting medium (Klinipath B.V., Duiven, The Netherlands) and covered with a cover slip. Slides were analyzed using light microscopy (Leica DMLB S/N:178295).

**FACS labeling**

Human renal cortex tissue from the unaffected pole of kidneys after tumor nephrectomy was cut into small pieces (≤1 mm) and further processed using enzymatic digestion and manual dissociation. In brief, small pieces of tissue were incubated in incubation medium consisting of DMEM/F12 (Gibco/Thermo Fisher Scientific, Breda, The Netherlands) supplemented with 200 U/ml collagenase IV (Thermo Fisher Scientific), 2 U/ml dispase II (Sigma-Aldrich), and 50 U/ml DNAse I (Sigma-Aldrich) for 15 min at 37 °C. This was pressed through a 150-µm sieve using the plunger of a syringe and washed with incubation medium. After this, the tissue solution was incubated again for 10 min in the incubation medium at 37 °C. This solution was rinsed through a 70- then a 32-µm sieve and washed using a saline solution. The flow-through was collected and centrifuged for 5 min at 1,500 rcf, the supernatant was removed, and the pellet was resuspended in NaCl solution. The isolated cell pellet was resuspended in 1 ml PBS containing 1% BSA (v/v, Sigma-Aldrich) and antibodies against CD133, CD24, and CD13 (supplementary material, Table S1) were used to stain proximal tubule epithelial cells (PTECs) and scattered tubular cells (STCs). Antibody incubation was performed for 1 h at 4 °C in the dark. The pellet was washed three times with PBS and centrifuged at 1,500 ×*g* for 5 min, the supernatant was removed, and the pellet was resuspended in PBS. This cell suspension was rinsed through a cell strainer (70-µm) and collected in a fluorescence-activated cell sorting (FACS) tube for cell sorting.

**FACS**

Cell sorting was performed to isolate PTECs and STCs for RNA sequencing using BD FACSAria instrument and software (BD Bioscience, Vianen, The Netherlands). PTECs were detected based on CD13 (PECy5)-positive staining, without CD24 (FITC) and CD133 (PE) staining. STCs were detected based on CD13 (PECy5) CD24 (FITC) and/or CD13 (PECy5) CD133 (PE) positive staining. For bulk RNA sequencing (100,000 cells per sample), cells were collected in Eppendorf tubes in PBS and RNA isolation was performed.

**Bulk RNA sequencing analysis**

Reads were trimmed, using TrimGalore! version 0.4.5, and mapped to a human reference genome (GRCh38.95, Ensembl) with Star version 2.6.0a. MultiQC was used to combine results and perform quality checks. Counts were produced with HTSeq version 0.11.0. Differential gene expression analysis was carried out with DESeq2 (version 1.32.0) in R (version 4.1.0), with internal statistical and normalization methods (i.e. multiple testing correction with Benjamini–Hochberg) [56]. The average mRNA expression of PTEC samples was compared to the average mRNA expression of STC samples. All heatmaps were created using the pheatmap package (version 1.0.12). Significantly (adj *P* value < 0.05) up- or down-regulated genes between PTEC samples and STC samples were used as input for overrepresentation analysis using ClusterProfiler (version 4.0.2) and the ReactomePA package (version 1.36.0) [57,58]. Pathway activity was analyzed using the PROGENy package (version 1.14.0) using the top 100 pathway “footprint genes” [59]. Next, transcription factor activity was analyzed using the DoRothEA package (version 1.4.1) using regulons with confidence levels: A, B, and C [60].

**Single-cell RNA sequencing analysis**

The publicly available human kidney dataset from a study by Muto *et al*. with accession number GSE151302 at the gene expression omnibus (GEO) was downloaded [29]. This dataset was processed using Seurat (version 4.0.6) in R. First, cells were filtered to keep only cells containing 500–4000 features with less than 20% of mitochondrial gene expression. Next, all datasets were merged using Seurat, and the following functions were performed with standard parameters: NormalizeData, FindVariableFeatures, ScaleData, and RunPCA. Next, harmony (version 0.1.0) integration of the datasets was performed, followed by the next steps in the Seurat workflow: FindNeighbors, FindClusters, and RunUMAP. Twenty-four principal components (PCs) were used to find neighbors and to run the UMAP, whereas clusters were found with a resolution of 0.7. We identified 20 clusters, and *VCAM1*-positive and -negative proximal tubule cells were identified by *SLC34A1* and *HAVCR1* expression based on the results in the study by Muto *et al*. (see supplementary material, Figure S2A,B). After isolation of the PT and PT_VCAM1 clusters, we excluded cells that were part of other clusters, which were named possible doublets and are shown in supplementary material, Figure S2C. Next, we performed a new UMAP projection using the RunUMAP function (supplementary material, Figure S2D). As for the bulk RNA dataset, we performed overrepresentation analysis using significant cluster markers with a positive log fold-change found by FindAllMarkers as input for the ClusterProfiler package. We also performed pathway analysis using PROGENy (using the top 500 “footprint genes”) and transcription factor activity analysis using DoRothEA (confidence levels A, B, and C) [61]. Additionally, we performed pseudotime analysis using Monocle3 (version 1.0.0) [62]. Before pseudotime analysis using Monocle3, the Seurat object was first converted to a cell dataset object, and subsequently we performed the standard workflow as provided by the laboratory of Cole Trapnell. Finally, ligand–receptor analysis was performed using CellphoneDB to calculate ligand–receptor interaction scores between clusters [63]. Counts and metadata were extracted from the Seurat object and saved as text files. The text files were used as input for the CellphoneDB (version 1) analysis in Ubuntu on Windows. After analysis, the output was processed and cell–cell interaction (CCI) plots and Sankey plots were created using the R package CrossTalkeR (version 1.2.0) [64].

**
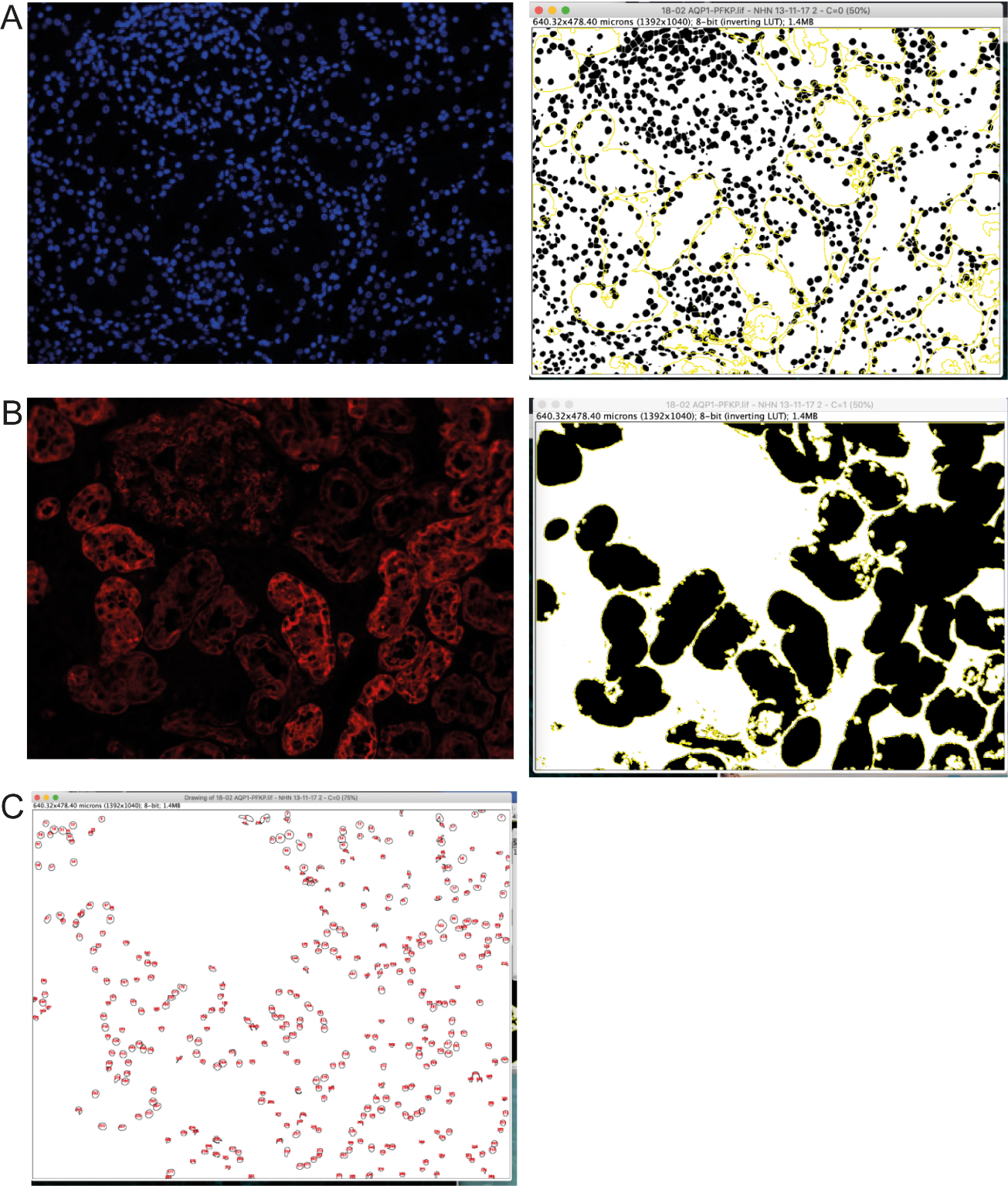
**

**Figure S1.** Fiji workflow for automated counting of proximal tubular epithelial cells (PTECs). (A) DAPI staining for all nuclei (blue). Using a threshold and a binary mask, all nuclei are shown as black dots (see right image) in ImageJ. (B) AQP-1 staining as a PTEC marker (red). An automated threshold was used and glomeruli were manually deleted. The tubular lumen was filled and whole tubules including the tubular lumen are shown in black in ImageJ (right image). (C) Using this selection, the nuclei only present inside the proximal tubule areas can be automatically counted using the ImageJ analysis tool. The workflow of this analysis per channel is summarized below (1. Workflow PTEC analysis). Abbreviations: AQP-1, aquaporin-1; PTEC, proximal tubular epithelial cell.

**1. Workflow PTEC analysis**

ImageJ, calculation of number of PTECs:

C0: DAPI

1.       Image - Adjust - Threshold: Black and white (B&W), Huang, dark background - apply

2.       Process - Binary - convert to mask

3.       Process - binary - watershed

C1: AQP1

1.       Image - adjust - threshold: B&W, Huang, dark background - apply

2.       Remove gloms by circling them using freehand polygon selection - backspace

3.       Process - binary - fill holes

4.       Edit - Selection - create selection

Click on C0:

1.       Shift + E

2.       Analyze – Analyze particles: size (10–500), show outlines, display results, clear results, summarize

**
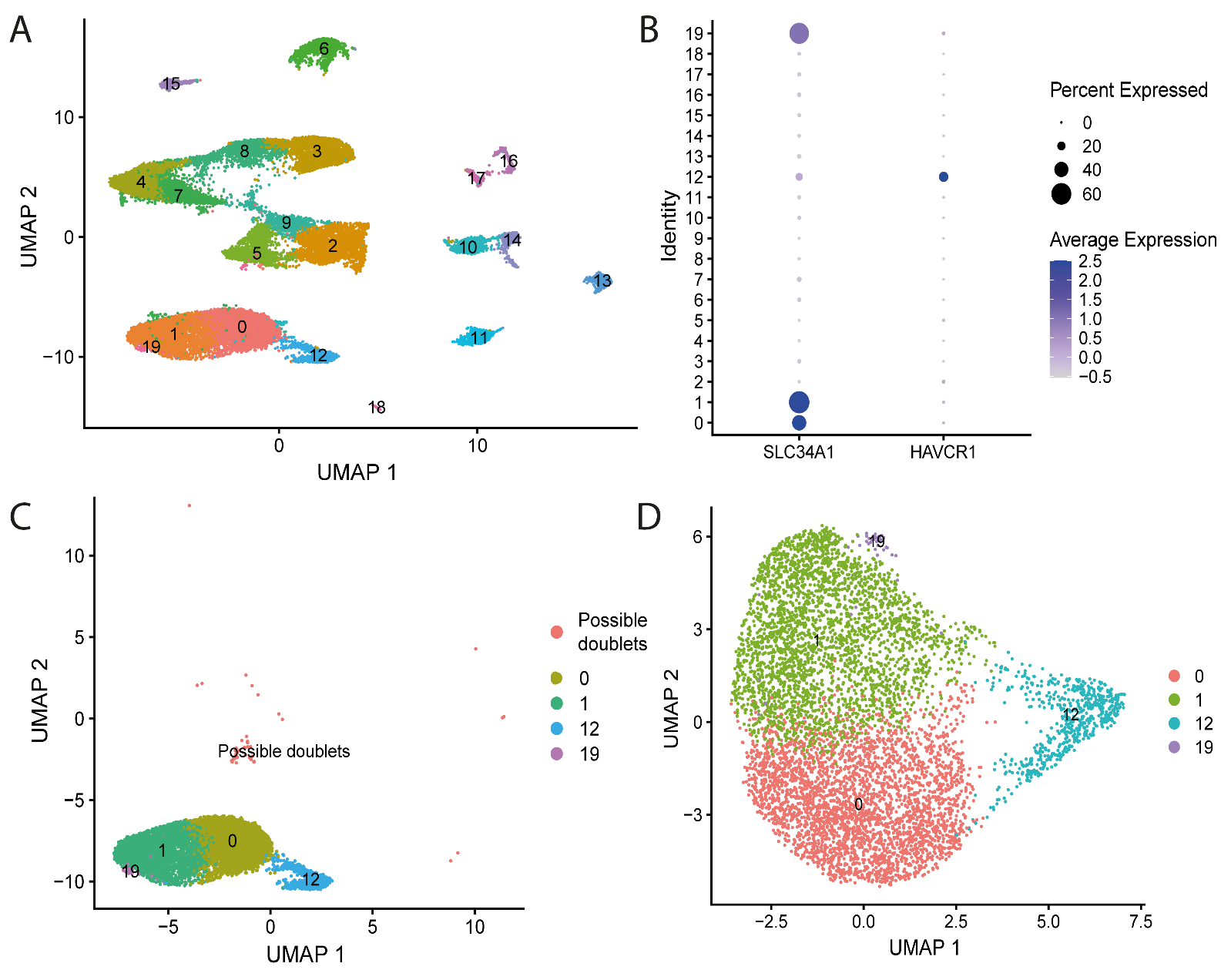
**

**Figure S2.** Preprocessing of single-cell sequencing database and subsequent clustering. (A) UMAP projection of five integrated single-cell RNA sequencing datasets of healthy human kidneys, downloaded from the online gene expression omnibus repository (Accession No. GSE151302). (B) DotPlot showing expression of *SLC34A1* and *HAVCR1* in clusters identified in human kidney. In the original manuscript, the authors identified the proximal tubule cluster and the PT_VCAM1 cluster using these two genes, and therefore we used a similar approach to separate the PT and PT_VCAM1 clusters from the entire kidney dataset. (C) We removed cells that belonged to the clusters that we separated from the dataset but were present in other cell clusters since these might be doublets belonging to two different clusters. (D) New UMAP projection after separating the PT and PT_VCAM1 clusters and removing the possible doublets. Abbreviations: UMAP, uniform manifold approximation and projection.


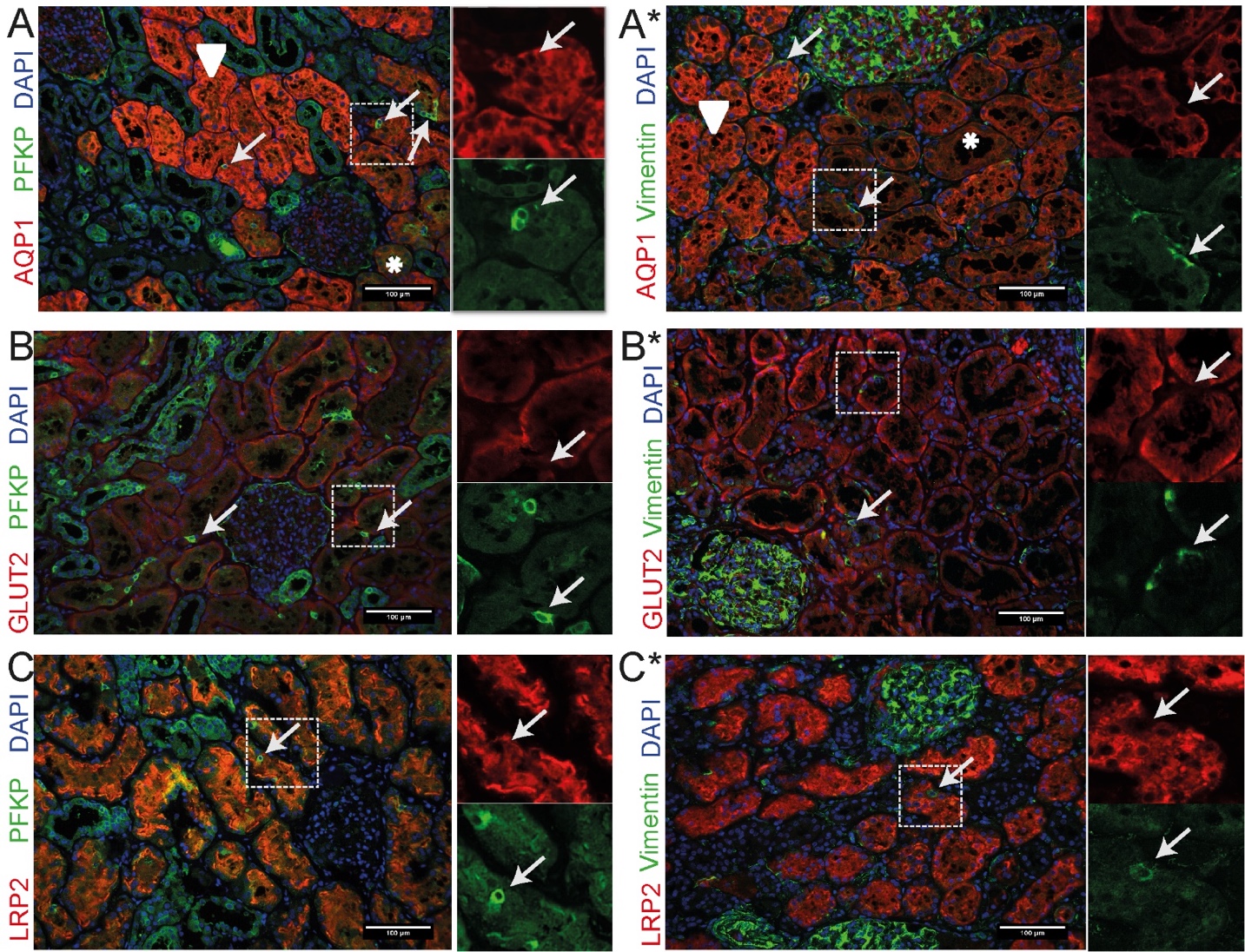


**Figure S3.** Scattered tubular cells are present in all segments of the proximal tubule. (A, A*). Immunofluorescence staining for AQP-1 (red) can be detected inside the proximal tubule, showing staining of different intensity with strong expression in proximal straight tubules (arrowheads) and less intense staining in the convoluted part of the proximal tubule (asterisk). In both parts of the proximal tubule, STCs (white arrows) can be detected, indicated by expression of PFKP (green, A) or vimentin (green, A*). (B, B*) Immunofluorescence staining for GLUT2 (red) as a proximal tubule marker, together with STC marker PFKP (green, B) or vimentin (green, B*) showing the presence of STCs (white arrows) inside the proximal tubule. (C, C*) Staining for megalin (LRP2) (red), which is expressed in proximal tubule segments S1 and S2. In the megalin positive proximal tubules, STCs (white arrows) can be detected as indicated by the expression of PFKP (green, C) and vimentin (green, C*). Scale bars: (A–C*) 100 µm. Abbreviations: AQP-1, aquaporin-1; GLUT2, glucose transporter 2; PFKP, phosphofructokinase-platelet; STC, scattered tubular cell.

**
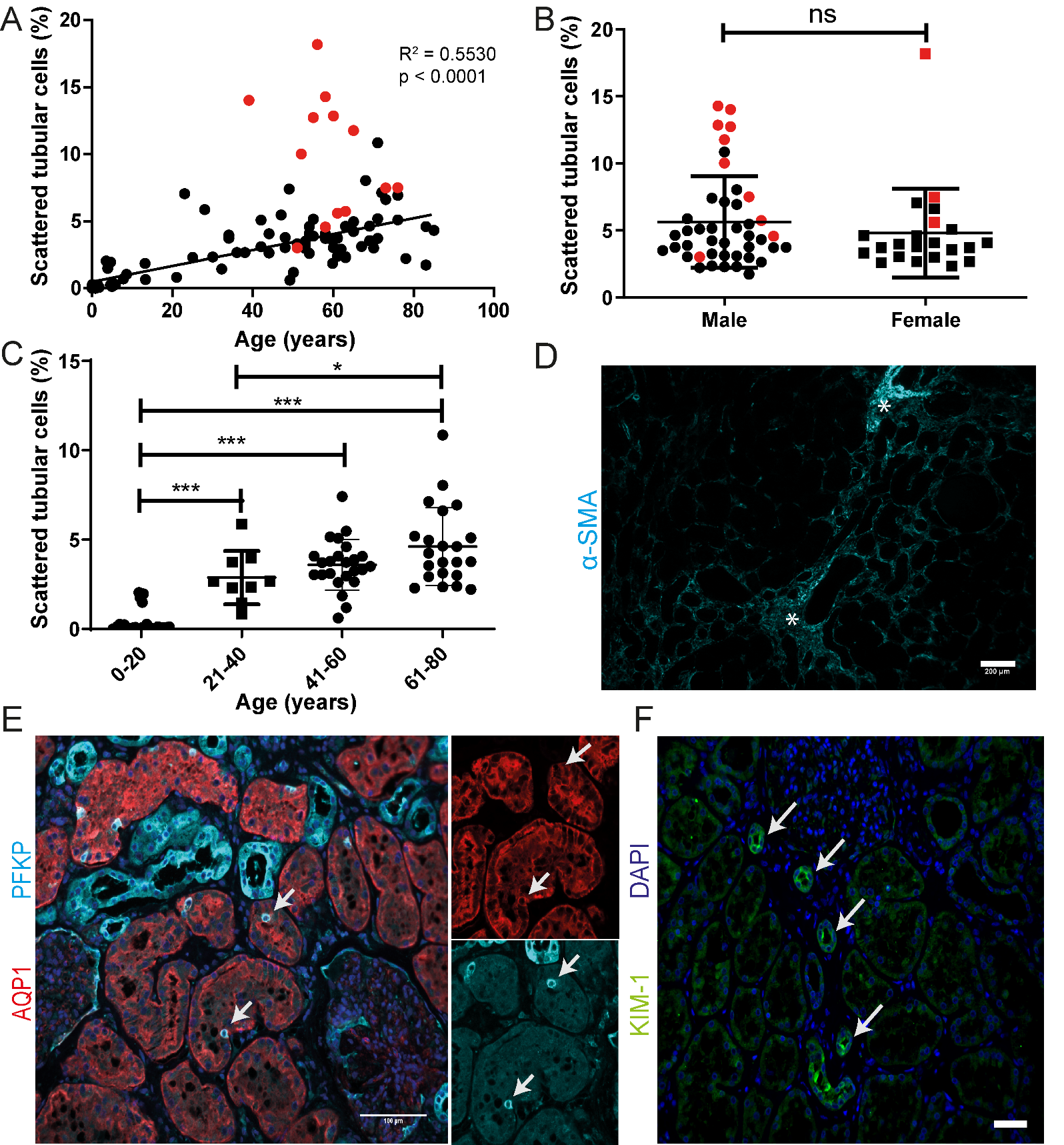
**

**Figure S4.** Scattered tubular cell numbers increase with age. (A) Regression analysis of STC number versus age was performed for normal human kidney tissue obtained from tumor nephrectomies using unaffected pole of 37 patients (48–85 years), 29 renal tissue samples obtained from renal transplant biopsies from donor kidneys prior to transplantation (age 23–76) and 41 young kidney tissues obtained from autopsy (age 0–61 years). The number of STCs significantly increases with age. The red points show patients with α-SMA expression of more than 5% of whole tissue slide surface area (significant deviation from zero excluding all tissues with more than 5% α-SMA expression (red points), *R*^2^ = 0.5530, *P* < 0.0001). (B) Analysis of the number of STCs in relation to gender showed no significant difference between male and female in normal human kidneys derived from unaffected pole after tumor nephrectomy. Kidneys with a high expression of α-SMA are indicated in red. (C) In clustering of age groups (child-young adults, adults, middle aged, and elderly), samples with a high α-SMA expression of more than 5% of whole tissue slide surface area are excluded. One-way ANOVA shows significant differences between age groups. (D) Representative microscopic image of immunofluorescence staining for α-SMA (cyan) as marker for interstitial fibrosis (asterisk). Surface area stained positive for α-SMA was calculated automatically in ImageJ. (E) Representative image of AQP1 staining as a PTEC marker (red) together with an STC marker PFKP (cyan) showing STCs (arrows) located inside a proximal tubule. With this staining, the percentage of PTECs that showed PFKP expression was calculated as percentage STCs present in kidneys. Analysis was performed on eight images per kidney (×20). (F) Representative image of immunofluorescence staining for KIM-1 (green) as a marker for acute tubular injury shows expression of KIM-1 in proximal tubules at luminal side (arrows). * = *P* < 0.05, *** = *P* < 0.001. Abbreviations: α-SMA, alpha-smooth muscle actin; AQP1, aquaporin-1; KIM-1, kidney injury molecule 1; PFKP, phosphofructokinase-platelet; PTEC, proximal tubular epithelial cell; STC, scattered tubular cell.

**2. Workflow α-SMA analysis in Image J**

ImageJ, Analysis aSMA expression surface area:

1.       Open lif file: view stack with hyperstack, color mode: default, split channels, autoscale.

2.       Make 8bit image

3.       Image – adjust – threshold – isodata, red, dark background - apply

4.       Analyze – set measurements – area, area fraction, limit to threshold, display label, mean gray value – ok

5.       Draw polygon selection

6.       Analyze – measure

**
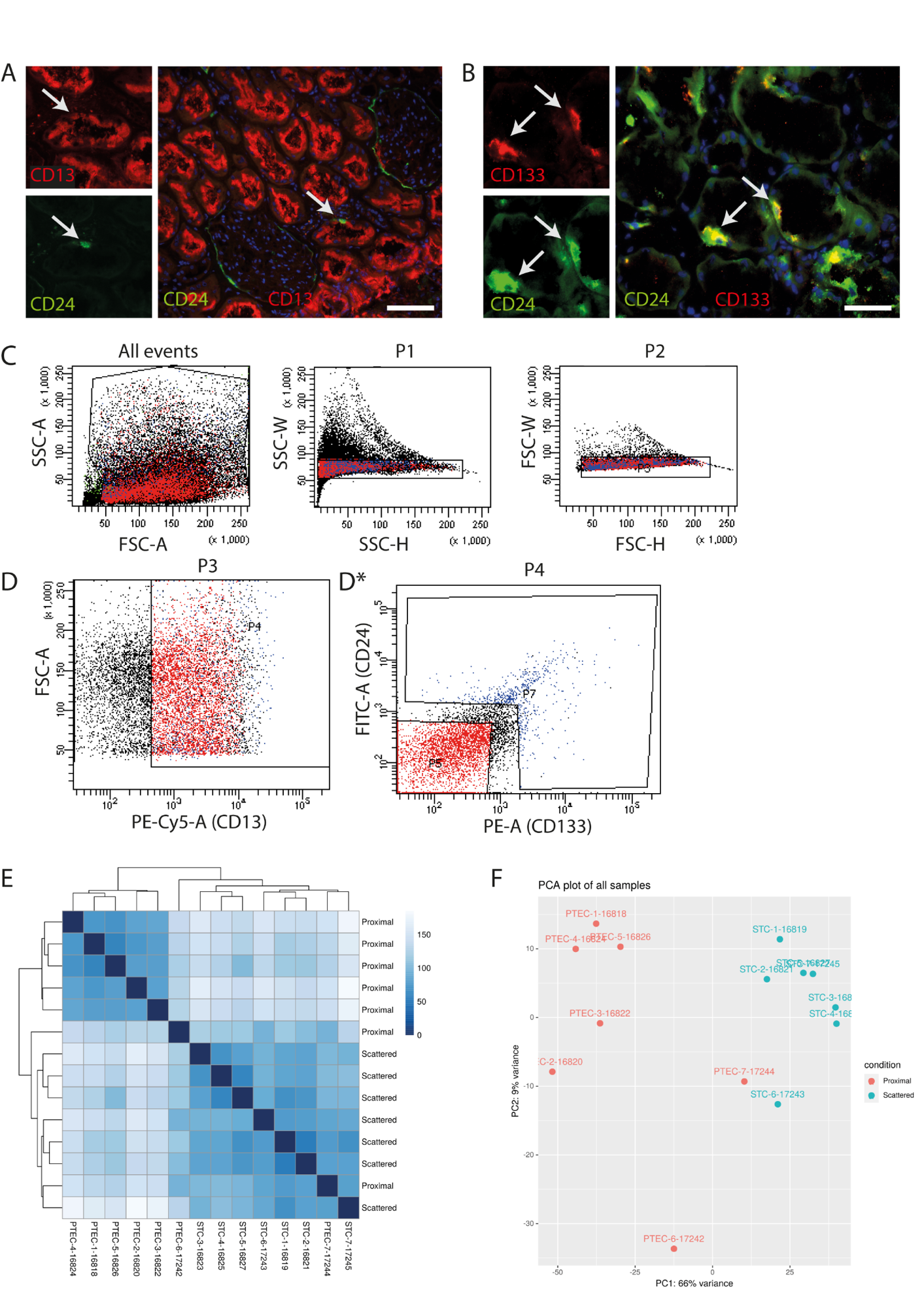
**

**Figure S5.** Fluorescence-activated cell sorting strategy and exploratory bulk RNA sequencing analysis. (A) Immunofluorescence staining for CD13 (red) as a marker for proximal tubule and CD24 (green) as a marker for STCs, shows STC expressing CD13 and CD24 (white arrow) inside a proximal tubule. (B) Representative image of immunofluorescence staining for CD24 and CD133, which colocalize in STCs (white arrow) and are not expressed by the other PTECs. (C–C**) Cell sorting using BD FACSAria to isolate STCs and PTECs. (C) Forward and side scatter gating. (D) P3 was selected based on forward and side scatter gating, and a new selection of cells was made based on cell size (FSC-A) versus CD13 expression (PE-Cy5). CD13-positive cells were selected in P4. (D*) All CD13-expressing cells were screened for expression of CD24 (FITC) and/or CD133 (PE). CD13-expressing cells negative for CD24 and CD133 were selected and sorted for PTEC analysis (P5). CD13-positive cells expressing CD24 and/or CD133 were selected and sorted for STC analysis (P7). (E) Exploratory data analysis of raw RNA-seq samples was performed using DESeq2 software packages. The Euclidean distances between the samples as calculated from the regularized log transformation are shown in the heatmap of the sample-to-sample distances. (F) PCA generated with ggplot2 library showing a PCA plot for all samples. Different colors denote the two conditions: orange = PTECs, blue = STCs. Scale bar: (A) 100 µm, (B) 50 µm. Abbreviations: PCA, principal component analysis; PTEC, proximal tubular epithelial cell; STC, scattered tubular cell.

**
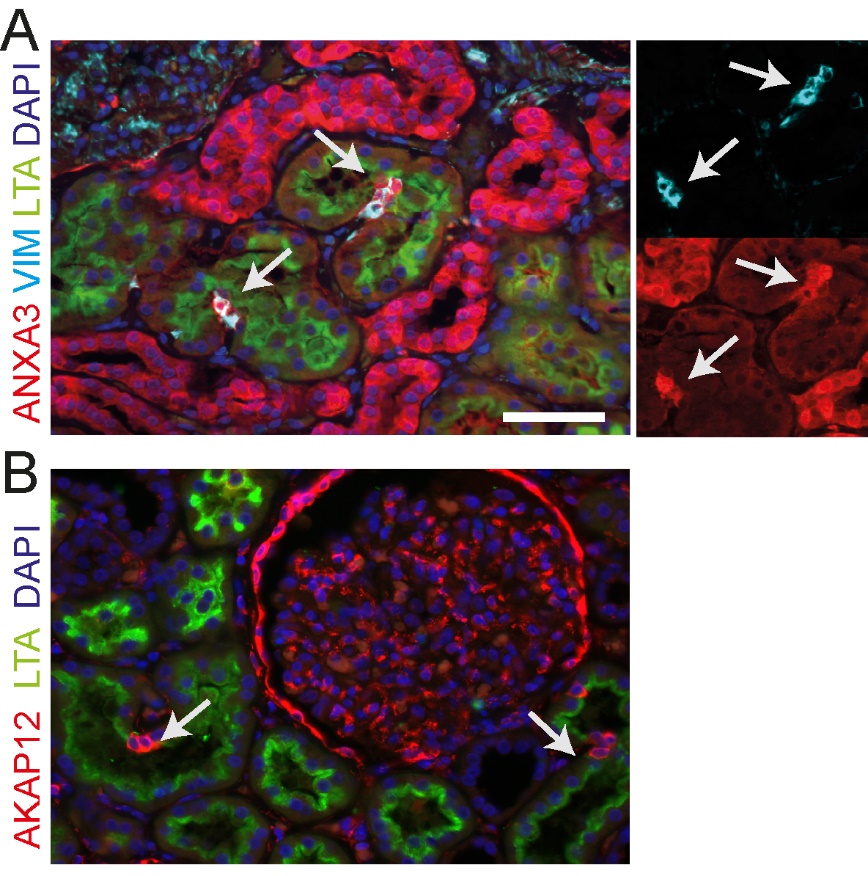
**

**Figure S6.** Immunohistochemical validation of STC markers upregulated in bulk RNA sequencing data. (A) Representative image of STCs (arrows) expressing vimentin (VIM, cyan) and annexin A3 (ANXA3, red), located in a proximal tubule (LTA-positive, green). (B) Immunofluorescence staining for AKAP12 (red) expressing STCs (arrows) inside proximal tubule (stained for LTA [green], which is located at the apical side). Scale bars: (A, B) 50 µm. Abbreviations: A-kinase anchoring protein 12; LTA, *Lotus tetragonolobus* lectin; STC, scattered tubular cell.

**
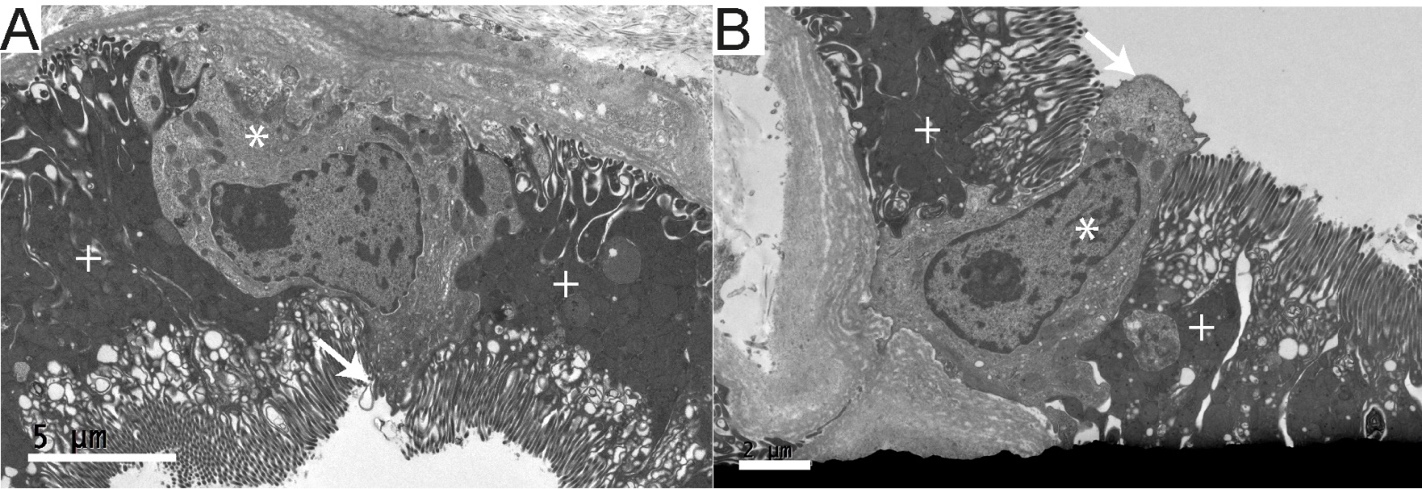
**

**Figure S7.** Scattered tubular cells show less mitochondria and a rudimentary brush border and basal labyrinth. (A, B) Representative images of STCs (asterisks) using transmission electron microscopy. Adjacent PTECs (plus) are characterized by an intact brush border and basal labyrinth and show a high density of mitochondria (dark gray), identifying them as PTECs. STCs show loss of brush border (arrow) and a rudimentary basal labyrinth and are less packed with mitochondria and other cell organelles. Scale bars: (A) 5 µm, (B) 2 µm.

**
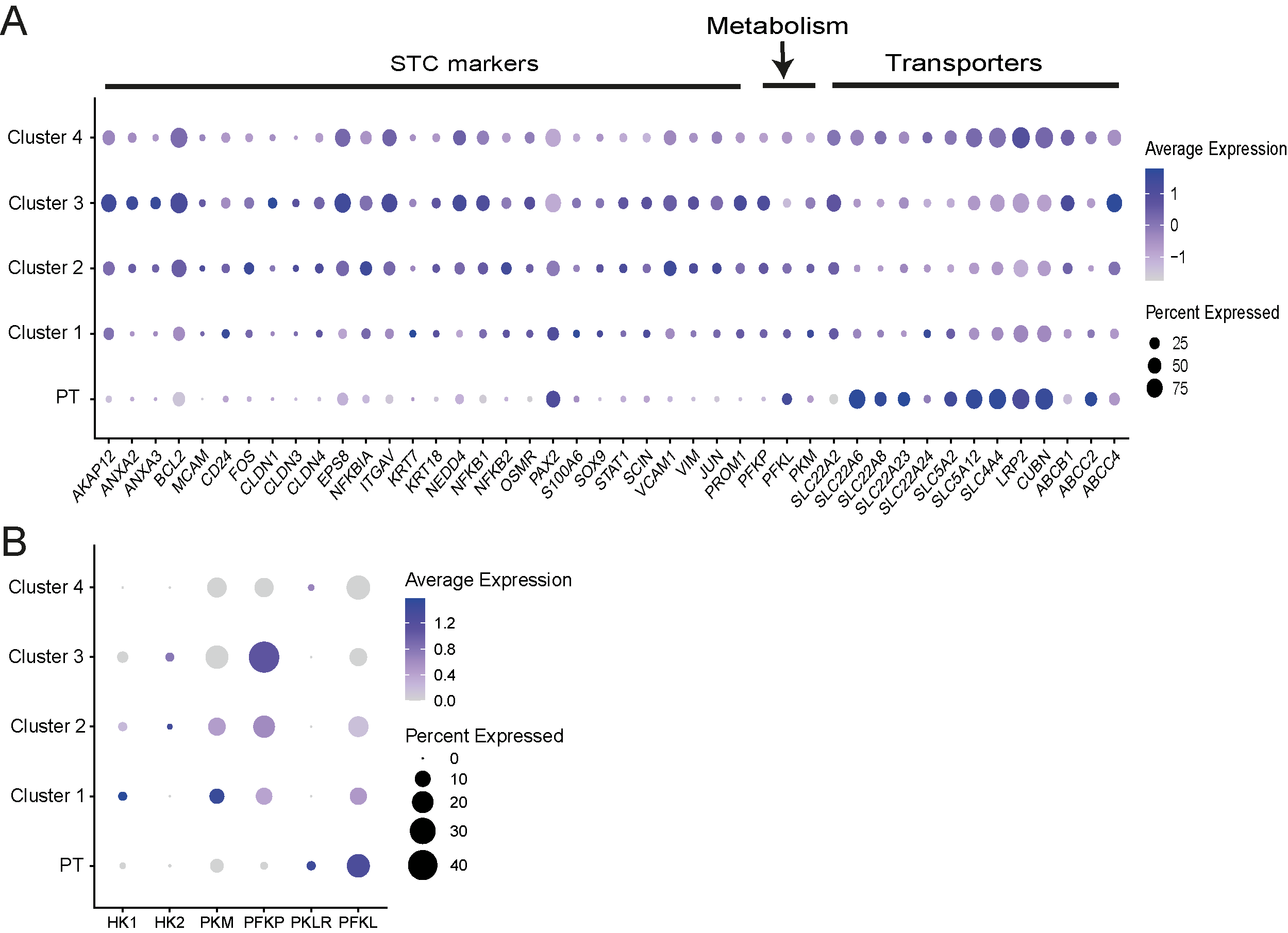
**

**Figure S8.** Scattered tubular cell clusters exhibit different gene expression profiles. (A) DotPlot showing relative gene expression of genes that are significantly associated with pseudotime. Cells are grouped by "STC markers", "Metabolism" or "Transporters" and indicate genes associated with STC phenotype, metabolic switch from oxidative phosphorylation to glycolysis or transporter function associated with normal PT functioning, respectively. STC markers increase nearly all in the PT_VCAM1 clusters compared to the PT cluster. Abbreviations: PT, proximal tubule; STC, scattered tubular cell.

**Supplementary Tables S1,S2**

**Table S1.** Primary antibodies used for immunofluorescence and immunohistochemical staining.

| **Primary antibody** | **Species** | **Dilution** | **Manufacturer** |
| --- | --- | --- | --- |
| α-smooth muscle actin (α-SMA) | Mouse | 1:500 | ab7817, Abcam, Cambridge, UK |
| Annexin A3 (ANXA3) | Rabbit | 1:100 | HPA013398, Sigma Aldrich, Zwijndrecht, The Netherlands |
| Annexin A2 (ANXA2) | Mouse | 1:200 | sc-28385, Santa Cruz Biotechnology, Dallas, TX, USA |
| Aquaporin 1 (AQP1) | Rabbit | 1:200 | #800765777, Alpha Diagnostics, San Antonio, TX, USA |
| Megaline/LRP2 | Rabbit | 1:100 | HPA064792, Sigma Aldrich |
| Glucose transporter 2 GLUT2/SLC2A2 | Rabbit | 1:100 | HPA028997, Sigma Aldrich |
| Phosphofructokinase, platelet, PFKP | Mouse | 1:100 | TA503983, Origene, Herford, Germany |
| P16, cyclin-dependent kinase inhibitor 2A, CDKN2A | Mouse | 1:100 | ILM0632-C01, Immunologic, Duiven, The Netherlands |
| Tumor suppressor p53 | Mouse | 1:100 | ILM27011-C01, Immunologic |
| Vimentin/V9 | Mouse | 1:100 | MA5-11883, Invitrogen/Thermo Fisher Scientific, Breda, The Netherlands |
| Vimentin | Rabbit | 1:100 | RM-9120-S1, Thermo Fisher Scientific, Breda, The Netherlands |
| Lamin B1, LMNB1 | Rabbit | 1:100 | HPA050524, Sigma Aldrich |
| A-kinase anchor protein 12, AKAP12 | Rabbit | 1:100 | TA322670, Origene |
| Pyruvate kinase PKL | Mouse | 1:100 | sc-1332222, Santa Cruz Biotechnology |
| Pyruvate kinase PKM2 | Rabbit | 1:100 | #3198, Cell Signaling Technology, Leiden, The Netherlands |
| Lotus Tetragonolobus lectin, LTA-FITC | Directly labeled | 1:200 | FL1321, Vector Laboratories, Peterborough, UK |
| aminopeptidase N, CD13-PECy5 | Directly labeled | 1:25 | A07763, Beckman Coulter, Woerden, The Netherlands |
| Prominin-1, CD133-PE | Directly labeled | 1:25 | #130-113-108, Miltenyi Biotec, Leiden, The Netherlands |
| Cluster of differentiation 24, CD24-FITC | Directly labeled | 1:25 | MHCD2401, Thermo Fisher Scientific |

**Table S2.** Secondary antibodies used for immunofluorescence and immunohistochemical staining.

| **Secondary antibody** | **Species** | **Dilution** | **Manufacturer** |
| --- | --- | --- | --- |
| Alexa Fluor 647 | Donkey-anti-mouse | 1:200 | ab150107, Abcam, Cambridge, UK |
| Alexa Fluor 647 | Goat-anti-rabbit | 1:200 | A-21245, Invitrogen/Thermo Fisher Scientific, Breda, the Netherlands |
| Alexa Fluor 568 | Goat-anti-rabbit | 1:200 | A-11036, Invitrogen |
| Alexa Fluor 569 | Donkey-anti-rabbit | 1:200 | A-10042, Invitrogen |
| Biotinylated | Horse-anti-mouse | 1:200 | BA2000, Vector laboratories, Peterborough, UK |
| Alexa Fluor 488 | Goat-anti-mouse | 1:200 | A-11029, Invitrogen |
| Alexa Fluor 488 | Donkey-anti-rabbit | 1:200 | A-21206, Invitrogen |
| Brightvision+ Poly-HRP | Goat-anti-mouse |  | DPVM110HRP, Immunologic, Duiven, the Netherlands |
| Brightvision+ Poly-HRP | Goat-anti-rabbit |  | DPVR55HRP, Immunologic |
